# Effects of two side-by-side camera trap deployments on estimations of richness, abundance, and the detection of medium- and large-sized mammals

**DOI:** 10.1371/journal.pone.0346028

**Published:** 2026-03-27

**Authors:** Sergio Guerrero-Vázquez, Silvia S. Zalapa, Salvador Mandujano, Ahtziri S. Basilio-Barrera, Marisela Pérez-Moreno

**Affiliations:** 1 Instituto de Zoología, Centro Universitario de Ciencias Biológicas y Agropecuarias, Universidad de Guadalajara, Zapopan, Jalisco, Mexico; 2 Red de Biología y Conservación de Vertebrados, Instituto de Ecología A. C., Xalapa, Veracruz, Mexico; 3 Posgrado BIMARENA, Centro Universitario de Ciencias Biológicas y Agropecuarias, Universidad de Guadalajara, Universidad de Guadalajara, Zapopan, Jalisco, Mexico; Sher-e Kashmir University of Agricultural Sciences and Technology of Jammu, INDIA

## Abstract

Medium- and large-sized mammals play key ecological roles but remain difficult to monitor due to low detectability and logistical constraints. Camera traps are widely used to address these challenges; however, survey outcomes are strongly influenced by sampling design. In this study, we evaluated how deploying two side-by-side camera traps per site, compared with a single-camera configuration, affects sampling efficiency and information gain in a small-scale monitoring grid. We quantified differences in detected species richness, number of photographic records, and time to first detection between single- and double-camera deployments. Using two cameras per site significantly increased detected species richness and the number of records per species and reduced time to first detection. Survival analyses showed that detection occurred more than twice as fast under the double-camera design, with particularly strong gains for rarely detected species. Comparisons with a conservative single-camera scenario further highlighted the risk of delayed or missed detections under limited sampling effort. Rather than testing novel ecological hypotheses, our results provide empirically grounded guidance on how site-level camera-trap deployment choices influence detection efficiency, inventory completeness, and the reliability of commonly used biodiversity metrics.

## Introduction

The study of medium- and large-sized mammals poses a central challenge in conservation ecology due to the environmental significance of this group and the difficulty of directly recording their presence in the field [1,2]. These organisms often occupy key positions in trophic networks as predators, prey, or seed dispersers, and their presence and abundance are considered indicators of ecosystem conservation status [3]. However, they have characteristics that complicate their study. Many species are nocturnal or cryptic, elusive, and occur at low population densities, moving across large home ranges, which makes direct observation methods largely ineffective [4]. In this context, camera traps have become a fundamental tool in biodiversity studies, particularly for terrestrial mammal research. These devices enable continuous photographic records of wildlife without capture and with minimal environmental disturbance [5]. Additionally, the images obtained provide verifiable evidence to facilitate taxonomic identification and offer further insights into the species’ behavior, activity patterns, habitat use, and interspecific interactions [6].

The main advantages of camera trapping include its minimally invasive nature, ability to operate autonomously for extended periods, and possibility of generating comparable datasets across sites and times [7]. For these reasons, camera trapping has become a standard method for monitoring programs that assess mammalian diversity across various habitat types [4,8]. However, camera traps have important limitations. One of the primary concerns is the imperfect detection probability [6,9,10]. Cameras record only individuals that pass within their detection zone, which is influenced by technical factors (sensor sensitivity, angle of view), environmental conditions (vegetation cover, topography), and biological traits (body size, behavior, and species density) [6,11,12]. These constraints can lead to an underestimation of species richness, biased relative abundance indices, and an incomplete representation of community composition [13].

Several studies have demonstrated that sampling designs relying on a single camera per site may underestimate richness and bias occupancy estimates, particularly for rare or cryptic species [14,15]. To address these limitations, an alternative methodological approach is to increase the number of cameras at each site. Deploying two cameras simultaneously provides a broader field of view and coverage across different directions or microhabitats, thereby increasing the probability of species detection, reducing the time required to record rare species, and improving estimates of key metrics, such as richness and relative abundance [10,12,16]. Most studies using paired camera traps have positioned them facing each other to enable bilateral photographic capture and individual identification, particularly of felids [17]. This configuration has historically focused on individual-based analyses, whereas, to our knowledge, no study has evaluated the use of two cameras placed side-by-side at the same site to improve detection probability and overall sampling efficiency at the community level. From the perspective of occupancy theory and detectability models, increasing sampling effort is directly associated with improved detection probability, thereby reducing biases in derived analyses [9].

In this study, we evaluated whether deploying two side-by-side camera traps per site represents a meaningful improvement over a single-camera design in terms of sampling efficiency and information gain. Specifically, we assessed how this design choice influences detected species richness, the number of photographic records, and time to first detection under a small-scale camera-trap grid implemented in a low-density system. By focusing on the magnitude and timing of detection gains, our objective was to quantify how increased sampling effort at the site level affects inventory completeness and the inclusion of rarely detected species, which remain critical challenges in applied monitoring programs. In this context, we hypothesized that deploying two side-by-side cameras per site would result in higher detected richness, more records per species, and shorter detection times, thereby providing empirically grounded guidance for camera-trap survey design under realistic logistical constraints.

## Materials and methods

### Study area

**Fig 1:**
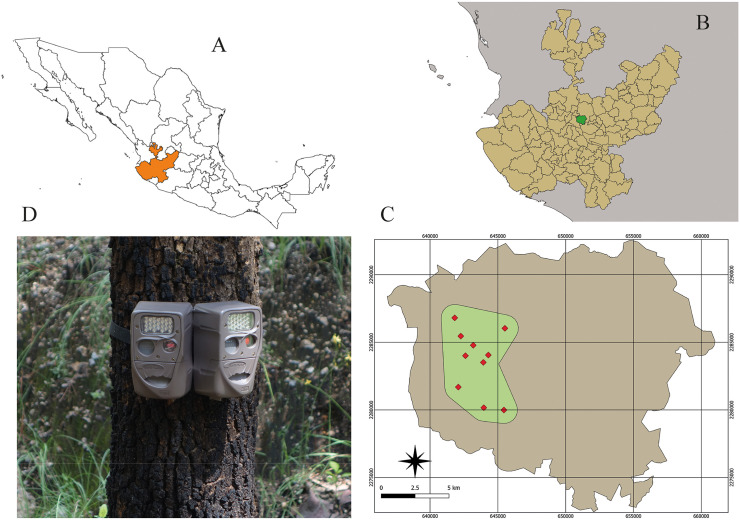
Location of the APFF La Primavera, Jalisco, Mexico; Sampling Sites and Camera Trap Arrangement. **Shapefiles obtained from the CONABIO geoportal (** **http://www.conabio.gob.mx/gis/** **).**

This study was conducted in the *Área de Protección de Flora y Fauna La Primavera* (APFF La Primavera), located in the state of Jalisco, western Mexico. The protected area spans 30,500 hectares and is located at the edge of the Guadalajara Metropolitan Area (ZMG), which comprises nine municipalities, including Guadalajara. La Primavera is of volcanic origin and is primarily covered by pine-oak, oak-pine, and tropical dry forests, as well as riparian vegetation. Several permanent and temporary streams occur within the reserve, particularly in the western sector. The area maintains a good conservation status despite its northeastern-to-southeastern boundary being adjacent to the most densely urbanized zone of the ZMG. Nevertheless, anthropogenic pressures, including wildfires, human settlements, human activities, and the presence of feral fauna (e.g., free-ranging dogs), have increased in recent years. Land tenure is a mosaic of communal (ejido), private, and state-owned properties, and the fieldwork for this study was conducted within the state-owned portion (approximately 7,000 ha; Fig 1A-C).

### Fieldwork

Field sampling was conducted from October 2024 to March 2025; fieldwork in the APFF La Primavera was conducted under authorization from the OPD La Primavera. From a long-term monitoring network of 26 camera-trap sites within the APFF La Primavera, we randomly selected 10 sites, maintaining a minimum distance of 1,000 m between them. At each site, two camera traps were installed side by side (Fig 1D) to expand the detection zones. The cameras were set at the same height (40–50 cm above ground) and at the same level to avoid performance bias. We used the Cuddeback H-1453 model. This trail camera features a motion sensor with a wide detection angle of approximately 50 degrees. At a distance of 10 meters, this angle translates to a horizontal detection width of approximately 9.5 meters. At each sampling site, we installed two camera traps oriented to have an angle between their optical axes of approximately 50–60°. Each camera was programmed to operate continuously (24 h) with a 15-second delay between triggers. The field teams visited the sites every 2–4 weeks to retrieve data and ensure that the devices functioned properly. The cameras were named camera A and camera B and remained fixed in the same position throughout the study.

### Data analysis

We defined three treatments for data analysis: camera A, camera B, and the double camera. For a single camera, successive photographic records of the same species at a given site were considered independent only if they were separated by at least 60 minutes; records occurring within shorter intervals were treated as a single event. When more than one individual of the same species appeared in a single photographic event, the record was counted as a single detection event, regardless of group size. Thus, the number of records reflects detection events rather than the number of individuals, consistent with standard practice in camera-trap studies aimed at estimating detection rates and relative abundance indices. Under the dual-camera setup, records from cameras A and B were merged into a single dataset, and the independence criteria previously defined for a single camera were applied. To test whether two cameras, compared with a single camera, increased species richness, we transformed the records into detection/ non-detection matrices for each species and site [18,19] and calculated species richness for each site and treatment. Pairwise comparisons were conducted using the one-tailed Wilcoxon signed-rank test, which is suitable for counting data without the assumption of normality [20]. We also estimated the median difference (Δ richness), Hodges–Lehmann estimator (HL) with 95% bootstrap confidence intervals, and rank-biserial effect size (rrb) [21]. Additionally, we constructed rarefaction and extrapolation curves [22,23] to compare the expected richness among treatments with equal sample size and coverage. To evaluate similarity in species composition between cameras, we calculated Jaccard similarity from detection/non-detection data and Bray-Curtis similarity from the number of records per species.

To assess whether the two cameras increased the number of records per species, we constructed a matrix of total records per species and site for each treatment. Paired analyses were conducted considering only the effective pairs (site-species combinations with data in both treatments). We applied one-tailed Wilcoxon signed-rank tests to assess whether the double-camera treatment yielded more records than the single-camera treatment. Confidence intervals were estimated for median differences, and rank-biserial effect sizes were calculated when applicable [21].

**Fig 2:**
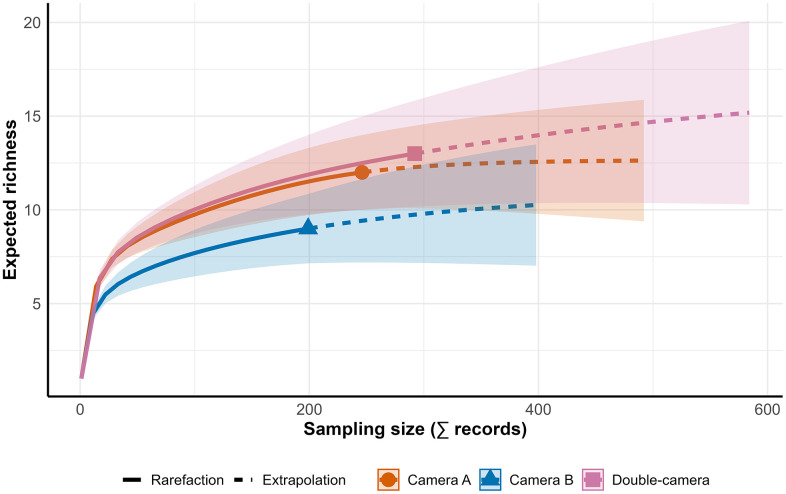
Rarefaction–Extrapolation Curve Showing the Relationship Between the Number of Records and Expected Species Richness.

We also analyzed the time to first detection for each species, defined as the interval between camera deployment and the first independent photograph at a site [11,18]. For each site-species-camera combination, we extracted the first detection date; if no detection occurred, we used the maximum operational period as the censoring time. We constructed two comparative scenarios to isolate the effect of camera deployment at the site level. In the single-camera scenario, time-to-first detection was conservatively defined as the longer time-to-detection (or censoring) between the two cameras at a site, representing a worst-case outcome in which only one camera is deployed and happens to perform sub-optimally. In contrast, the double-camera scenario was defined as the shorter time-to-detection, corresponding to detection by either camera. This paired site-level comparison allows both scenarios to be evaluated under identical environmental and temporal conditions, while avoiding confounding effects arising from site heterogeneity. Survival analyses were used to estimate the cumulative probability of non-detection over time (S(t)) [24]. Kaplan–Meier curves [25] were fitted for each scenario and compared using log-rank tests [26]. Cox proportional hazards models [27] were fitted, with the single-camera scenario as the reference, such that hazard ratios (HR) > 1 indicate faster detection under the double-camera design. This analysis was not intended to compare the performance of individual cameras, which is addressed elsewhere (e.g., Fig 2), but to evaluate site-level survey outcomes under alternative deployment designs. To evaluate rare species and provide guidelines for sampling duration, we calculated the time (Tp) required to reach cumulative detection probabilities of p = 0.8 and p = 0.95, respectively. Rare species were defined as those within the lower quartile (25%) of the total number of detected events [28,29]. The Tp values were estimated from the quantiles of species- and treatment-specific survival functions.

All analyses were conducted in R v. 4.4.1 [30] using the *survival* [28] and *iNEXT* [31] packages.

## Results

A total of 445 independent records (246 from camera A and 199 from camera B) were obtained over 3,600 trap days (180 days per camera). Across both cameras, 13 medium- and large-sized mammal species were recorded: 12 species with camera A and 9 with camera B (Table 1). The species similarity between the cameras was 0.61 according to the Jaccard index and 0.85 according to the Bray-Curtis similarity index.

**Table 1:** List of Species Recorded and Number of Independent Records by Treatment During the Camera-Trap Survey Conducted in APFF La Primavera, Jalisco, Mexico.

Species	Camera A	Camera B	Double-camera
*Bassariscus astutus*	2	1	3
*Canis familiaris*	23	13	25
*Canis latrans*	2	2	3
*Conepatus leuconotus*	11	0	11
*Dasypus novemcinctus*	0	1	1
*Dicotyles angulatus*	76	80	90
*Didelphis virginiana*	1	0	1
*Lynx rufus*	24	19	26
*Mephitis macroura*	2	0	2
*Nasua narica*	27	32	39
*Odocoileus virginianus*	58	47	74
*Procyon lotor*	1	0	1
*Urocyon cinereoargenteus*	19	4	22
TOTAL	246	199	298

Paired comparisons indicated that, compared with a single camera, using two cameras per site increased the recorded species richness. Across the 10 sites, the mean richness with the double-camera treatment (5.8 species) was greater than that with camera A (5.2 species; HL = 0.5; 95% CI: 0–1; p = 0.044) and camera B (4.3 species; HL = 1.5; 95% CI: 1–2.5; p = 0.006). In both cases, the effect sizes were large (rrb = 1). These results demonstrate that deploying two cameras per site increases the detected species richness.

Rarefaction and extrapolation curves showed consistent differences among the sampling treatments (Fig 2). The estimated richness was always higher with the double-camera design, reaching values close to 15 species, whereas approximately 12 and 9 species were registered with camera A and camera B, respectively. This pattern was evident in both sample size and coverage-based estimates (SC ≈ 0.99), indicating that the double-camera treatment increased the observed richness. Camera A performed moderately well and was relatively close to the double-camera estimates, whereas camera B markedly underestimated the community richness. These findings suggest that using two cameras instead of one significantly increases the detected richness, improves the sampling coverage, and provides more robust biodiversity estimates.

In terms of the number of photographic records per species, the double-camera treatment consistently yielded more records than the single-camera treatment (Table 1). Differences were more pronounced between the double camera (mean = 5.03 records) and camera B (mean = 3.43 records; HL = 2.00; p < 0.0001) than between the double camera and camera A (mean = 4.24 records; HL = 1.50; p < 0.0001).

Comparisons between the double-camera design and a scenario using only the least efficient camera at each site revealed consistent differences in detection (S1 Table). Among the 58 evaluated records, the double-camera treatment always detected the species, whereas the least efficient camera missed 18 cases (31%). In cases where both species were detected (69%), the double-camera treatment detected them on average 45 days earlier (median = 15 days), with a maximum advantage of 169 days. In 64% of the cases, the double camera detected species earlier, and in no case did a single camera detect species first. These results demonstrate that the double-camera design not only increases detection probability but also significantly reduces the time to first detection, thereby minimizing biases associated with suboptimal single-camera performance.

**Fig 3:**
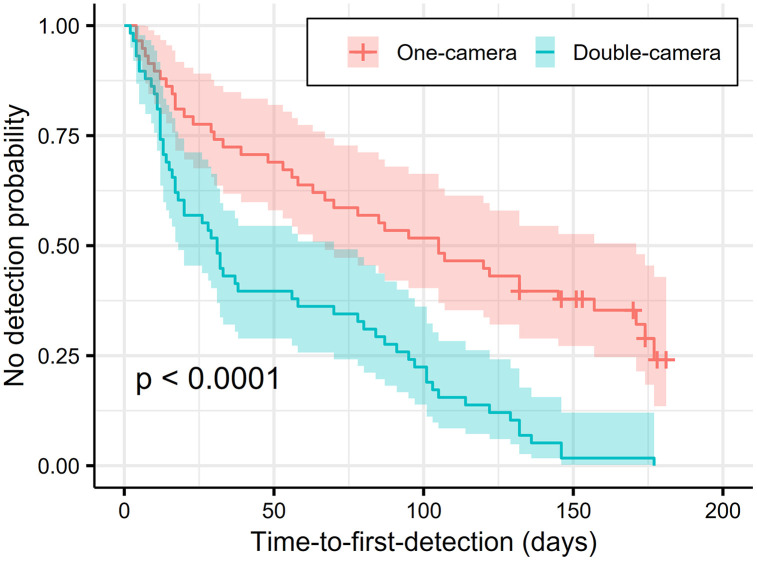
Kaplan–Meier Curves of Time-to-First-Detection for Single-Camera Versus Double-Camera Treatments, with a Log-Rank Test.

The Kaplan–Meier curves clearly differed between treatments, indicating contrasting detection dynamics over time. The probability of non-detection decreased more slowly under the single-camera treatment than under the double-camera treatment, reflecting delayed detection when sampling effort was lower (Fig 3). These results demonstrate that survival-type analyses provide a useful framework for quantifying temporal differences in detection efficiency associated with alternative camera-trap configurations. The log-rank test confirmed significant differences between the groups (χ² = 17.2, df = 1, p < 0.0001), indicating that the use of two cameras reduced the time to first detection.

Cox proportional hazards models adjusted by species and clustered by site further revealed that the double-camera treatment was associated with a higher hazard of detection than the single-camera treatment (HR = 2.62; 95% CI: 1.85–3.71; p < 0.0001). This finding indicates that species were detected more quickly when two cameras were deployed, corroborating the Kaplan–Meier results. Proportionality diagnostics revealed no violations (p = 0.64), validating the use of the Cox model.

We identified five rare species, defined as those in the lower quartile (<25%) of the total number of detected events. For these species, detection-time analyses revealed that the time required to reach cumulative detection probabilities of 0.8 and 0.95 was consistently shorter under the double-camera treatment than under the single-camera treatment. To achieve a detection probability > 0.8, the required time was 87 days with two cameras, compared with 157 days with one camera. Moreover, in the single-camera treatment, four of the five rare species never reached estimable detection times, whereas in the double-camera treatment, all the species were detected within reasonable timeframes. These findings highlight the clear advantage of deploying two side-by-side cameras to detect uncommon species within shorter sampling periods.

## Discussion

Our results consistently show that using two side-by-side camera traps per site improves sampling performance compared with using a single camera trap, thereby confirming our initial hypothesis. In terms of species richness, we observed significant increases across comparisons, indicating that single-camera designs may underestimate diversity. These findings align with those of previous studies that reported limitations due to restricted fields of view when only one camera was used [11,13,14]. The use of Kaplan–Meier curves allowed us to compare the temporal dynamics of detection between camera-trap configurations using a non-parametric and distribution-free framework. Because both treatments were deployed simultaneously across spatially independent stations, differences in the probability of non-detection can be attributed to sampling effort rather than temporal variation in species activity. While Kaplan–Meier analyses do not explicitly account for imperfect detection or site-level covariates, they provide a transparent and informative way to evaluate how quickly species accumulate under different field designs. In this context, the marked divergence between treatments demonstrates the utility of survival-type analyses for identifying efficiency gains that are not captured by richness or count metrics alone. To our knowledge, this approach has rarely been applied to evaluate camera-trap configurations in community-level studies, underscoring its value as a complementary tool for optimizing sampling design.

Several studies have used two or more cameras per site to increase detection probability by covering different directions or microhabitats, and paired-camera configurations have frequently been implemented for bilateral identification in species such as felids [13,16,17]. These approaches differ conceptually from our design, in which both cameras are positioned side by side on the same support with the explicit purpose of widening the field of view at a single sampling point. By expanding the effective detection cone without altering site density or spatial replication, the side-by-side configuration isolates the functional contribution of field-of-view expansion rather than simply increasing the number of deployed cameras. Interpreted in light of this distinction, the gains we observed in species richness and time to first detection reflect the effect of enhanced visual coverage, underscoring the methodological novelty and practical value of this deployment strategy for community-level camera-trap surveys.

The number of photographic records was greater in the double side-by-side camera treatment. This methodological gain was consistent and associated with large effect sizes, supporting the idea that greater sampling effort increases the probability of recording individuals and activity events [6,12]. From a practical standpoint, using two cameras per site can increase the reliability of local detection metrics and relative abundance indices without expanding the number of sampling sites. However, we acknowledge the well-established trade-off between increasing sampling effort within sites and increasing spatial replication across sites. Expanding the number of sites can better capture spatial heterogeneity, include a wider range of habitat types, and increase the likelihood of detecting species with specialized habitat requirements or large home ranges [4,10,29]. In addition, species that live in groups or move in close succession may exhibit higher detection rates, particularly under double-camera deployments [13]. Although this effect could contribute to higher detection rates for gregarious species, detection events in this study were defined independently of group size, and our analyses focus on relative comparisons between deployment designs rather than on absolute abundance. In this context, double-camera deployments should not be viewed as a substitute for broader spatial coverage, but rather as a complementary strategy that improves detection efficiency and inventory completeness when logistical, financial, or access constraints limit the number of sites that can be sampled [6,16]. The optimal balance between site replication and within-site sampling effort ultimately depends on study objectives and ecological context, a topic widely discussed in the camera-trapping literature [13,32].

An important applied contribution of this study is the use of time-to-first detection as an operational metric to evaluate sampling efficiency across alternative camera-trap designs. Rather than proposing a novel survival modeling framework, we apply well-established survival analyses to quantify how quickly information is gained under single- versus double-camera deployments [24,25,27]. In this context, the single-camera scenario was intentionally defined using the least efficient camera at each site as a conservative, worst-case benchmark, representing the outcome of deploying only one camera that happens to perform suboptimally. This paired, site-level approach allows the effect of deployment design to be isolated under identical environmental and temporal conditions, without conflating design effects with camera-specific performance. From the perspective of detectability theory, detection is inherently imperfect and strongly influenced by sampling effort and design choices [9,33]. Our results show that doubling camera effort at the site level substantially accelerates detection, reduces censoring, and improves the inclusion of rare species within shorter sampling periods, patterns consistent with previous findings linking increased effort to higher detection probability [6,16]. From a survey design perspective, these gains are critical because delayed or missed detections directly affect inventory completeness and the reliability of downstream analyses, particularly in short-term or resource-limited monitoring programs [29,32]. Taken together, our findings confirm that using two cameras increases species richness and the number of records while optimizing the balance between field effort and information return, thereby enabling faster and more reliable detection of rare or sensitive species. This evidence reinforces the importance of incorporating double-camera designs into conservation programs that aim to monitor medium- and large-sized mammal communities in a timely manner [4,34].

Nevertheless, some limitations and considerations must be acknowledged. Increasing the number of cameras per site entails additional acquisition, maintenance, and logistical costs [7,8]. Moreover, the magnitude of the benefit may depend on ecological factors, such as population density, home range size, and habitat characteristics [35,36]. Future research should assess whether the observed benefits are consistent across different ecosystems and whether there are density or diversity thresholds at which a single camera may suffice.

In conclusion, deploying two camera traps per site represents a robust and practical strategy for improving sampling efficiency in camera-trap surveys of medium- and large-sized mammals. By increasing detection probability, accelerating time to first detection, and ensuring the inclusion of rarely detected species, the double-camera design enhances inventory completeness without increasing the number of sampling sites. Although this approach entails additional logistical and economic costs, our results show that these costs translate into substantial information gains, particularly under short sampling periods or constrained monitoring frameworks. This study contributes to current debates on how site-level design decisions influence the quality, timing, and reliability of data obtained in wildlife studies using camera traps, and thus, decision-making for their conservation.

## Supporting information

S1 Table Data Used to Test Time-to-First-Detection, Obtained During the Camera-Trap Survey Conducted in APFF La Primavera, Jalisco, Mexico. (DOCX)

S2 Table Data with Records and Presence, for Each Site a Species, Obtained During the Camera-Trap Survey Conducted in APFF La Primavera, Jalisco, Mexico. (DOCX)
