# Supplementary material for: Effects of two side-by-side camera trap deployments on estimations of richness, abundance, and the detection of medium- and large-sized mammals
Source: PLoS One. 2026 Mar 27;21(3):e0346028. doi: 10.1371/journal.pone.0346028 (PMC13028507; doi:10.1371/journal.pone.0346028)
Supplement: S1 Table — (DOCX) [file pone.0346028.s001.docx]

**S1 Table. Data Used to Test Time-to-First-Detection, Obtained During the Camera-Trap Survey Conducted in APFF La Primavera, Jalisco, Mexico.**

| Site | Species | Group | Camera | Start_Cam | End_Cam | Time | Detection |
| --- | --- | --- | --- | --- | --- | --- | --- |
| F06 | *Dasypus novemcinctus* | OneWorst | A | 01/10/2024 | 03/03/2025 | 153 | 0 |
| F06 | *Conepatus leuconotus* | OneWorst | B | 01/10/2024 | 01/03/2025 | 151 | 0 |
| F06 | *Dicotyles angulatus* | OneWorst | B | 01/10/2024 | 01/03/2025 | 87 | 1 |
| F06 | *Odocoileus virginianus* | OneWorst | B | 01/10/2024 | 01/03/2025 | 151 | 0 |
| F06 | *Canis familiaris* | OneWorst | B | 01/10/2024 | 01/03/2025 | 39 | 1 |
| F06 | *Urocyon cinereoargenteus* | OneWorst | B | 01/10/2024 | 01/03/2025 | 63 | 1 |
| F06 | *Bassariscus astutus* | OneWorst | B | 01/10/2024 | 01/03/2025 | 151 | 0 |
| G09 | *Nasua narica* | OneWorst | A | 01/10/2024 | 02/04/2025 | 107 | 1 |
| G09 | *Odocoileus virginianus* | OneWorst | A | 01/10/2024 | 02/04/2025 | 4 | 1 |
| G09 | *Dicotyles angulatus* | OneWorst | A | 01/10/2024 | 02/04/2025 | 6 | 1 |
| G09 | *Lynx rufus* | OneWorst | A | 01/10/2024 | 02/04/2025 | 105 | 1 |
| G09 | *Canis familiaris* | OneWorst | A | 01/10/2024 | 02/04/2025 | 177 | 1 |
| H06 | *Dicotyles angulatus* | OneWorst | A | 01/10/2024 | 21/03/2025 | 16 | 1 |
| H06 | *Odocoileus virginianus* | OneWorst | A | 01/10/2024 | 21/03/2025 | 10 | 1 |
| H06 | *Canis familiaris* | OneWorst | A | 01/10/2024 | 21/03/2025 | 70 | 1 |
| H06 | *Nasua narica* | OneWorst | A | 01/10/2024 | 21/03/2025 | 78 | 1 |
| H06 | *Didelphis virginiana* | OneWorst | A | 01/10/2024 | 21/03/2025 | 157 | 1 |
| H06 | *Conepatus leuconotus* | OneWorst | B | 01/10/2024 | 24/02/2025 | 146 | 0 |
| H06 | *Urocyon cinereoargenteus* | OneWorst | B | 01/10/2024 | 24/02/2025 | 146 | 0 |
| H06 | *Canis latrans* | OneWorst | A | 01/10/2024 | 21/03/2025 | 171 | 1 |
| H07 | *Dicotyles angulatus* | OneWorst | B | 01/10/2024 | 20/03/2025 | 8 | 1 |
| H07 | *Odocoileus virginianus* | OneWorst | B | 01/10/2024 | 20/03/2025 | 85 | 1 |
| H07 | *Lynx rufus* | OneWorst | A | 01/10/2024 | 29/03/2025 | 17 | 1 |
| H07 | *Urocyon cinereoargenteus* | OneWorst | B | 01/10/2024 | 20/03/2025 | 120 | 1 |
| H07 | *Procyon lotor* | OneWorst | B | 01/10/2024 | 20/03/2025 | 170 | 0 |
| H07 | *Canis familiaris* | OneWorst | A | 01/10/2024 | 29/03/2025 | 20 | 1 |
| H07 | *Nasua narica* | OneWorst | A | 01/10/2024 | 29/03/2025 | 67 | 1 |
| I06 | *Odocoileus virginianus* | OneWorst | A | 01/10/2024 | 24/03/2025 | 174 | 1 |
| I06 | *Canis familiaris* | OneWorst | B | 01/10/2024 | 24/03/2025 | 174 | 0 |
| I06 | *Dicotyles angulatus* | OneWorst | A | 01/10/2024 | 24/03/2025 | 7 | 1 |
| I06 | *Mephitis macroura* | OneWorst | B | 01/10/2024 | 24/03/2025 | 174 | 0 |
| I06 | *Nasua narica* | OneWorst | A | 01/10/2024 | 24/03/2025 | 174 | 0 |
| I08 | *Odocoileus virginianus* | OneWorst | A | 01/10/2024 | 26/03/2025 | 95 | 1 |
| I08 | *Dicotyles angulatus* | OneWorst | A | 01/10/2024 | 26/03/2025 | 29 | 1 |
| I08 | *Nasua narica* | OneWorst | A | 01/10/2024 | 26/03/2025 | 14 | 1 |
| I08 | *Conepatus leuconotus* | OneWorst | B | 01/10/2024 | 20/03/2025 | 170 | 0 |
| I08 | *Canis familiaris* | OneWorst | B | 01/10/2024 | 20/03/2025 | 170 | 0 |
| I08 | *Canis latrans* | OneWorst | A | 01/10/2024 | 26/03/2025 | 132 | 1 |
| I08 | *Lynx rufus* | OneWorst | A | 01/10/2024 | 26/03/2025 | 17 | 1 |
| J08 | *Nasua narica* | OneWorst | A | 01/10/2024 | 31/03/2025 | 56 | 1 |
| J08 | *Odocoileus virginianus* | OneWorst | A | 01/10/2024 | 31/03/2025 | 48 | 1 |
| J08 | *Dicotyles angulatus* | OneWorst | A | 01/10/2024 | 31/03/2025 | 4 | 1 |
| J08 | *Lynx rufus* | OneWorst | A | 01/10/2024 | 31/03/2025 | 105 | 1 |
| J08 | *Canis latrans* | OneWorst | A | 01/10/2024 | 31/03/2025 | 181 | 0 |
| J08 | *Mephitis macroura* | OneWorst | B | 01/10/2024 | 31/03/2025 | 181 | 0 |
| L06 | *Dicotyles angulatus* | OneWorst | A | 01/10/2024 | 28/03/2025 | 12 | 1 |
| L06 | *Odocoileus virginianus* | OneWorst | A | 01/10/2024 | 28/03/2025 | 53 | 1 |
| L06 | *Nasua narica* | OneWorst | A | 01/10/2024 | 28/03/2025 | 178 | 0 |
| L06 | *Bassariscus astutus* | OneWorst | A | 01/10/2024 | 28/03/2025 | 178 | 0 |
| L06 | *Lynx rufus* | OneWorst | A | 01/10/2024 | 28/03/2025 | 178 | 0 |
| M08 | *Odocoileus virginianus* | OneWorst | A | 01/10/2024 | 23/02/2025 | 23 | 1 |
| M08 | *Dicotyles angulatus* | OneWorst | A | 01/10/2024 | 23/02/2025 | 33 | 1 |
| M08 | *Nasua narica* | OneWorst | A | 01/10/2024 | 23/02/2025 | 58 | 1 |
| M08 | *Conepatus leuconotus* | OneWorst | A | 01/10/2024 | 23/02/2025 | 145 | 1 |
| M09 | *Conepatus leuconotus* | OneWorst | B | 01/10/2024 | 10/02/2025 | 132 | 0 |
| M09 | *Odocoileus virginianus* | OneWorst | B | 01/10/2024 | 10/02/2025 | 30 | 1 |
| M09 | *Nasua narica* | OneWorst | A | 01/10/2024 | 10/02/2025 | 122 | 1 |
| M09 | *Dicotyles angulatus* | OneWorst | A | 01/10/2024 | 10/02/2025 | 132 | 1 |
| F06 | *Dasypus novemcinctus* | DobleCamera | A_or_B | 01/10/2024 | 03/03/2025 | 87 | 1 |
| F06 | *Conepatus leuconotus* | DobleCamera | A_or_B | 01/10/2024 | 03/03/2025 | 15 | 1 |
| F06 | *Dicotyles angulatus* | DobleCamera | A_or_B | 01/10/2024 | 03/03/2025 | 5 | 1 |
| F06 | *Odocoileus virginianus* | DobleCamera | A_or_B | 01/10/2024 | 03/03/2025 | 12 | 1 |
| F06 | *Canis familiaris* | DobleCamera | A_or_B | 01/10/2024 | 03/03/2025 | 32 | 1 |
| F06 | *Urocyon cinereoargenteus* | DobleCamera | A_or_B | 01/10/2024 | 03/03/2025 | 11 | 1 |
| F06 | *Bassariscus astutus* | DobleCamera | A_or_B | 01/10/2024 | 03/03/2025 | 129 | 1 |
| G09 | *Nasua narica* | DobleCamera | A_or_B | 01/10/2024 | 02/04/2025 | 32 | 1 |
| G09 | *Odocoileus virginianus* | DobleCamera | A_or_B | 01/10/2024 | 02/04/2025 | 4 | 1 |
| G09 | *Dicotyles angulatus* | DobleCamera | A_or_B | 01/10/2024 | 02/04/2025 | 3 | 1 |
| G09 | *Lynx rufus* | DobleCamera | A_or_B | 01/10/2024 | 02/04/2025 | 105 | 1 |
| G09 | *Canis familiaris* | DobleCamera | A_or_B | 01/10/2024 | 02/04/2025 | 177 | 1 |
| H06 | *Dicotyles angulatus* | DobleCamera | A_or_B | 01/10/2024 | 21/03/2025 | 16 | 1 |
| H06 | *Odocoileus virginianus* | DobleCamera | A_or_B | 01/10/2024 | 21/03/2025 | 10 | 1 |
| H06 | *Canis familiaris* | DobleCamera | A_or_B | 01/10/2024 | 21/03/2025 | 70 | 1 |
| H06 | *Nasua narica* | DobleCamera | A_or_B | 01/10/2024 | 21/03/2025 | 78 | 1 |
| H06 | *Didelphis virginiana* | DobleCamera | A_or_B | 01/10/2024 | 21/03/2025 | 146 | 1 |
| H06 | *Conepatus leuconotus* | DobleCamera | A_or_B | 01/10/2024 | 21/03/2025 | 31 | 1 |
| H06 | *Urocyon cinereoargenteus* | DobleCamera | A_or_B | 01/10/2024 | 21/03/2025 | 114 | 1 |
| H06 | *Canis latrans* | DobleCamera | A_or_B | 01/10/2024 | 21/03/2025 | 146 | 1 |
| H07B | *Dicotyles angulatus* | DobleCamera | A_or_B | 01/10/2024 | 29/03/2025 | 5 | 1 |
| H07B | *Odocoileus virginianus* | DobleCamera | A_or_B | 01/10/2024 | 29/03/2025 | 31 | 1 |
| H07B | *Lynx rufus* | DobleCamera | A_or_B | 01/10/2024 | 29/03/2025 | 17 | 1 |
| H07B | *Urocyon cinereoargenteus* | DobleCamera | A_or_B | 01/10/2024 | 29/03/2025 | 101 | 1 |
| H07B | *Procyon lotor* | DobleCamera | A_or_B | 01/10/2024 | 29/03/2025 | 95 | 1 |
| H07B | *Canis familiaris* | DobleCamera | A_or_B | 01/10/2024 | 29/03/2025 | 20 | 1 |
| H07B | *Nasua narica* | DobleCamera | A_or_B | 01/10/2024 | 29/03/2025 | 26 | 1 |
| I06 | *Odocoileus virginianus* | DobleCamera | A_or_B | 01/10/2024 | 24/03/2025 | 97 | 1 |
| I06 | *Canis familiaris* | DobleCamera | A_or_B | 01/10/2024 | 24/03/2025 | 20 | 1 |
| I06 | *Dicotyles angulatus* | DobleCamera | A_or_B | 01/10/2024 | 24/03/2025 | 7 | 1 |
| I06 | *Mephitis macroura* | DobleCamera | A_or_B | 01/10/2024 | 24/03/2025 | 12 | 1 |
| I06 | *Nasua narica* | DobleCamera | A_or_B | 01/10/2024 | 24/03/2025 | 13 | 1 |
| I08 | *Odocoileus virginianus* | DobleCamera | A_or_B | 01/10/2024 | 26/03/2025 | 84 | 1 |
| I08 | *Dicotyles angulatus* | DobleCamera | A_or_B | 01/10/2024 | 26/03/2025 | 29 | 1 |
| I08 | *Nasua narica* | DobleCamera | A_or_B | 01/10/2024 | 26/03/2025 | 14 | 1 |
| I08 | *Conepatus leuconotus* | DobleCamera | A_or_B | 01/10/2024 | 26/03/2025 | 37 | 1 |
| I08 | *Canis familiaris* | DobleCamera | A_or_B | 01/10/2024 | 26/03/2025 | 136 | 1 |
| I08 | *Canis latrans* | DobleCamera | A_or_B | 01/10/2024 | 26/03/2025 | 132 | 1 |
| I08 | *Lynx rufus* | DobleCamera | A_or_B | 01/10/2024 | 26/03/2025 | 17 | 1 |
| J08B | *Nasua narica* | DobleCamera | A_or_B | 01/10/2024 | 31/03/2025 | 56 | 1 |
| J08B | *Odocoileus virginianus* | DobleCamera | A_or_B | 01/10/2024 | 31/03/2025 | 38 | 1 |
| J08B | *Dicotyles angulatus* | DobleCamera | A_or_B | 01/10/2024 | 31/03/2025 | 4 | 1 |
| J08B | *Lynx rufus* | DobleCamera | A_or_B | 01/10/2024 | 31/03/2025 | 101 | 1 |
| J08B | *Canis latrans* | DobleCamera | A_or_B | 01/10/2024 | 31/03/2025 | 103 | 1 |
| J08B | *Mephitis macroura* | DobleCamera | A_or_B | 01/10/2024 | 31/03/2025 | 28 | 1 |
| L06 | *Dicotyles angulatus* | DobleCamera | A_or_B | 01/10/2024 | 28/03/2025 | 12 | 1 |
| L06 | *Odocoileus virginianus* | DobleCamera | A_or_B | 01/10/2024 | 28/03/2025 | 12 | 1 |
| L06 | *Nasua narica* | DobleCamera | A_or_B | 01/10/2024 | 28/03/2025 | 11 | 1 |
| L06 | *Bassariscus astutus* | DobleCamera | A_or_B | 01/10/2024 | 28/03/2025 | 9 | 1 |
| L06 | *Lynx rufus* | DobleCamera | A_or_B | 01/10/2024 | 28/03/2025 | 13 | 1 |
| M08 | *Odocoileus virginianus* | DobleCamera | A_or_B | 01/10/2024 | 23/02/2025 | 18 | 1 |
| M08 | *Dicotyles angulatus* | DobleCamera | A_or_B | 01/10/2024 | 23/02/2025 | 33 | 1 |
| M08 | *Nasua narica* | DobleCamera | A_or_B | 01/10/2024 | 23/02/2025 | 58 | 1 |
| M08 | *Conepatus leuconotus* | DobleCamera | A_or_B | 01/10/2024 | 23/02/2025 | 80 | 1 |
| M09 | *Conepatus leuconotus* | DobleCamera | A_or_B | 01/10/2024 | 10/02/2025 | 91 | 1 |
| M09 | *Odocoileus virginianus* | DobleCamera | A_or_B | 01/10/2024 | 10/02/2025 | 2 | 1 |
| M09 | *Nasua narica* | DobleCamera | A_or_B | 01/10/2024 | 10/02/2025 | 122 | 1 |
| M09 | *Dicotyles angulatus* | DobleCamera | A_or_B | 01/10/2024 | 10/02/2025 | 132 | 1 |
