# Supplementary material for: Effects of two side-by-side camera trap deployments on estimations of richness, abundance, and the detection of medium- and large-sized mammals
Source: PLoS One. 2026 Mar 27;21(3):e0346028. doi: 10.1371/journal.pone.0346028 (PMC13028507; doi:10.1371/journal.pone.0346028)
Supplement: S2 Table — (DOCX) [file pone.0346028.s002.docx]

**S2 Table. Data with Records and Presence, for Each Site a Species, Obtained During the Camera-Trap Survey Conducted in APFF La Primavera, Jalisco, Mexico.**

| Site | Treatment | Species | Records | Presence |
| --- | --- | --- | --- | --- |
| F06 | Camera_A | *Bassariscus astutus* | 2 | 1 |
| F06 | Camera_A | *Canis familiaris* | 8 | 1 |
| F06 | Camera_A | *Conepatus leuconotus* | 2 | 1 |
| F06 | Camera_A | *Dicotyles angulatus* | 5 | 1 |
| F06 | Camera_A | *Odocoileus virginianus* | 5 | 1 |
| F06 | Camera_A | *Urocyon cinereoargenteus* | 16 | 1 |
| F06 | Camera_B | *Canis familiaris* | 5 | 1 |
| F06 | Camera_B | *Dasypus novemcinctus* | 1 | 1 |
| F06 | Camera_B | *Dicotyles angulatus* | 1 | 1 |
| F06 | Camera_B | *Urocyon cinereoargenteus* | 2 | 1 |
| G09 | Camera_A | *Canis familiaris* | 1 | 1 |
| G09 | Camera_A | *Dicotyles angulatus* | 3 | 1 |
| G09 | Camera_A | *Lynx rufus* | 1 | 1 |
| G09 | Camera_A | *Nasua narica* | 2 | 1 |
| G09 | Camera_A | *Odocoileus virginianus* | 9 | 1 |
| G09 | Camera_B | *Canis familiaris* | 1 | 1 |
| G09 | Camera_B | *Dicotyles angulatus* | 3 | 1 |
| G09 | Camera_B | *Lynx rufus* | 1 | 1 |
| G09 | Camera_B | *Nasua narica* | 4 | 1 |
| G09 | Camera_B | *Odocoileus virginianus* | 8 | 1 |
| H06 | Camera_A | *Canis familiaris* | 4 | 1 |
| H06 | Camera_A | *Canis latrans* | 1 | 1 |
| H06 | Camera_A | *Conepatus leuconotus* | 1 | 1 |
| H06 | Camera_A | *Dicotyles angulatus* | 5 | 1 |
| H06 | Camera_A | *Didelphis virginiana* | 1 | 1 |
| H06 | Camera_A | *Nasua narica* | 3 | 1 |
| H06 | Camera_A | *Odocoileus virginianus* | 7 | 1 |
| H06 | Camera_A | *Urocyon cinereoargenteus* | 1 | 1 |
| H06 | Camera_B | *Canis familiaris* | 3 | 1 |
| H06 | Camera_B | *Dicotyles angulatus* | 3 | 1 |
| H06 | Camera_B | *Nasua narica* | 3 | 1 |
| H06 | Camera_B | *Odocoileus virginianus* | 5 | 1 |
| H07 | Camera_A | *Canis familiaris* | 6 | 1 |
| H07 | Camera_A | *Dicotyles angulatus* | 34 | 1 |
| H07 | Camera_A | *Lynx rufus* | 20 | 1 |
| H07 | Camera_A | *Nasua narica* | 3 | 1 |
| H07 | Camera_A | *Odocoileus virginianus* | 3 | 1 |
| H07 | Camera_A | *Procyon lotor* | 1 | 1 |
| H07 | Camera_A | *Urocyon cinereoargenteus* | 2 | 1 |
| H07 | Camera_B | *Canis familiaris* | 4 | 1 |
| H07 | Camera_B | *Dicotyles angulatus* | 33 | 1 |
| H07 | Camera_B | *Lynx rufus* | 14 | 1 |
| H07 | Camera_B | *Nasua narica* | 1 | 1 |
| H07 | Camera_B | *Odocoileus virginianus* | 2 | 1 |
| H07 | Camera_B | *Urocyon cinereoargenteus* | 2 | 1 |
| I06 | Camera_A | *Canis familiaris* | 3 | 1 |
| I06 | Camera_A | *Dicotyles angulatus* | 1 | 1 |
| I06 | Camera_A | *Mephitis macroura* | 1 | 1 |
| I06 | Camera_A | *Odocoileus virginianus* | 1 | 1 |
| I06 | Camera_B | *Dicotyles angulatus* | 1 | 1 |
| I06 | Camera_B | *Nasua narica* | 1 | 1 |
| I06 | Camera_B | *Odocoileus virginianus* | 2 | 1 |
| I08 | Camera_A | *Canis familiaris* | 1 | 1 |
| I08 | Camera_A | *Canis latrans* | 1 | 1 |
| I08 | Camera_A | *Conepatus leuconotus* | 1 | 1 |
| I08 | Camera_A | *Dicotyles angulatus* | 1 | 1 |
| I08 | Camera_A | *Lynx rufus* | 2 | 1 |
| I08 | Camera_A | *Nasua narica* | 8 | 1 |
| I08 | Camera_A | *Odocoileus virginianus* | 7 | 1 |
| I08 | Camera_B | *Canis latrans* | 1 | 1 |
| I08 | Camera_B | *Dicotyles angulatus* | 2 | 1 |
| I08 | Camera_B | *Lynx rufus* | 1 | 1 |
| I08 | Camera_B | *Nasua narica* | 7 | 1 |
| I08 | Camera_B | *Odocoileus virginianus* | 11 | 1 |
| J08 | Camera_A | *Dicotyles angulatus* | 14 | 1 |
| J08 | Camera_A | *Lynx rufus* | 1 | 1 |
| J08 | Camera_A | *Mephitis macroura* | 1 | 1 |
| J08 | Camera_A | *Nasua narica* | 7 | 1 |
| J08 | Camera_A | *Odocoileus virginianus* | 10 | 1 |
| J08 | Camera_B | *Canis latrans* | 1 | 1 |
| J08 | Camera_B | *Dicotyles angulatus* | 22 | 1 |
| J08 | Camera_B | *Lynx rufus* | 2 | 1 |
| J08 | Camera_B | *Nasua narica* | 12 | 1 |
| J08 | Camera_B | *Odocoileus virginianus* | 7 | 1 |
| L06 | Camera_A | *Dicotyles angulatus* | 10 | 1 |
| L06 | Camera_A | *Odocoileus virginianus* | 5 | 1 |
| L06 | Camera_B | *Bassariscus astutus* | 1 | 1 |
| L06 | Camera_B | *Dicotyles angulatus* | 12 | 1 |
| L06 | Camera_B | *Lynx rufus* | 1 | 1 |
| L06 | Camera_B | *Nasua narica* | 1 | 1 |
| L06 | Camera_B | *Odocoileus virginianus* | 4 | 1 |
| M08 | Camera_A | *Conepatus leuconotus* | 1 | 1 |
| M08 | Camera_A | *Dicotyles angulatus* | 2 | 1 |
| M08 | Camera_A | *Nasua narica* | 3 | 1 |
| M08 | Camera_A | *Odocoileus virginianus* | 4 | 1 |
| M08 | Camera_B | *Dicotyles angulatus* | 2 | 1 |
| M08 | Camera_B | *Nasua narica* | 2 | 1 |
| M08 | Camera_B | *Odocoileus virginianus* | 4 | 1 |
| M09 | Camera_A | *Conepatus leuconotus* | 6 | 1 |
| M09 | Camera_A | *Dicotyles angulatus* | 1 | 1 |
| M09 | Camera_A | *Nasua narica* | 1 | 1 |
| M09 | Camera_A | *Odocoileus virginianus* | 7 | 1 |
| M09 | Camera_B | *Dicotyles angulatus* | 1 | 1 |
| M09 | Camera_B | *Nasua narica* | 1 | 1 |
| M09 | Camera_B | *Odocoileus virginianus* | 4 | 1 |
| F06 | DobleCamara | *Bassariscus astutus* | 2 | 1 |
| F06 | DobleCamara | *Canis familiaris* | 10 | 1 |
| F06 | DobleCamara | *Conepatus leuconotus* | 2 | 1 |
| F06 | DobleCamara | *Dasypus novemcinctus* | 1 | 1 |
| F06 | DobleCamara | *Dicotyles angulatus* | 6 | 1 |
| F06 | DobleCamara | *Odocoileus virginianus* | 5 | 1 |
| F06 | DobleCamara | *Urocyon cinereoargenteus* | 18 | 1 |
| G09 | DobleCamara | *Canis familiaris* | 1 | 1 |
| G09 | DobleCamara | *Dicotyles angulatus* | 4 | 1 |
| G09 | DobleCamara | *Lynx rufus* | 1 | 1 |
| G09 | DobleCamara | *Nasua narica* | 4 | 1 |
| G09 | DobleCamara | *Odocoileus virginianus* | 10 | 1 |
| H06 | DobleCamara | *Canis familiaris* | 4 | 1 |
| H06 | DobleCamara | *Canis latrans* | 1 | 1 |
| H06 | DobleCamara | *Conepatus leuconotus* | 1 | 1 |
| H06 | DobleCamara | *Dicotyles angulatus* | 5 | 1 |
| H06 | DobleCamara | *Didelphis virginiana* | 1 | 1 |
| H06 | DobleCamara | *Nasua narica* | 3 | 1 |
| H06 | DobleCamara | *Odocoileus virginianus* | 7 | 1 |
| H06 | DobleCamara | *Urocyon cinereoargenteus* | 1 | 1 |
| H07B | DobleCamara | *Canis familiaris* | 6 | 1 |
| H07B | DobleCamara | *Dicotyles angulatus* | 36 | 1 |
| H07B | DobleCamara | *Lynx rufus* | 20 | 1 |
| H07B | DobleCamara | *Nasua narica* | 4 | 1 |
| H07B | DobleCamara | *Odocoileus virginianus* | 3 | 1 |
| H07B | DobleCamara | *Procyon lotor* | 1 | 1 |
| H07B | DobleCamara | *Urocyon cinereoargenteus* | 3 | 1 |
| I06 | DobleCamara | *Canis familiaris* | 3 | 1 |
| I06 | DobleCamara | *Dicotyles angulatus* | 1 | 1 |
| I06 | DobleCamara | *Mephitis macroura* | 1 | 1 |
| I06 | DobleCamara | *Nasua narica* | 1 | 1 |
| I06 | DobleCamara | *Odocoileus virginianus* | 2 | 1 |
| I08 | DobleCamara | *Canis familiaris* | 1 | 1 |
| I08 | DobleCamara | *Canis latrans* | 1 | 1 |
| I08 | DobleCamara | *Conepatus leuconotus* | 1 | 1 |
| I08 | DobleCamara | *Dicotyles angulatus* | 2 | 1 |
| I08 | DobleCamara | *Lynx rufus* | 2 | 1 |
| I08 | DobleCamara | *Nasua narica* | 9 | 1 |
| I08 | DobleCamara | *Odocoileus virginianus* | 12 | 1 |
| J08B | DobleCamara | *Canis latrans* | 1 | 1 |
| J08B | DobleCamara | *Dicotyles angulatus* | 22 | 1 |
| J08B | DobleCamara | *Lynx rufus* | 2 | 1 |
| J08B | DobleCamara | *Mephitis macroura* | 1 | 1 |
| J08B | DobleCamara | *Nasua narica* | 12 | 1 |
| J08B | DobleCamara | *Odocoileus virginianus* | 12 | 1 |
| L06 | DobleCamara | *Bassariscus astutus* | 1 | 1 |
| L06 | DobleCamara | *Dicotyles angulatus* | 11 | 1 |
| L06 | DobleCamara | *Lynx rufus* | 1 | 1 |
| L06 | DobleCamara | *Nasua narica* | 1 | 1 |
| L06 | DobleCamara | *Odocoileus virginianus* | 6 | 1 |
| M08 | DobleCamara | *Conepatus leuconotus* | 1 | 1 |
| M08 | DobleCamara | *Dicotyles angulatus* | 2 | 1 |
| M08 | DobleCamara | *Nasua narica* | 3 | 1 |
| M08 | DobleCamara | *Odocoileus virginianus* | 6 | 1 |
| M09 | DobleCamara | *Conepatus leuconotus* | 6 | 1 |
| M09 | DobleCamara | *Dicotyles angulatus* | 1 | 1 |
| M09 | DobleCamara | *Nasua narica* | 1 | 1 |
| M09 | DobleCamara | *Odocoileus virginianus* | 6 | 1 |
